# Supplementary figures and images for: Female Genital Schistosomiasis (FGS) in Cameroon: A formative epidemiological and socioeconomic investigation in eleven rural fishing communities
Source: PLOS Glob Public Health. 2021 Oct 20;1(10):e0000007. doi: 10.1371/journal.pgph.0000007 (PMC10022362; doi:10.1371/journal.pgph.0000007)

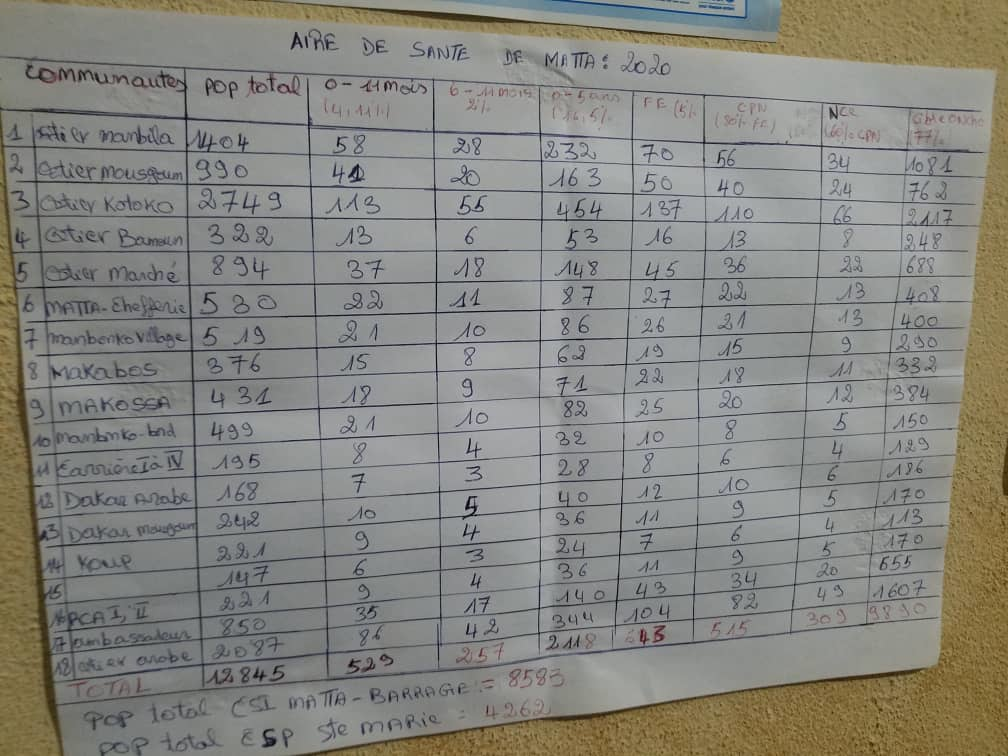

Supplement: S1 Fig — (TIF) [file pgph.0000007.s001.tif]
