# Supplementary material for: Female Genital Schistosomiasis (FGS) in Cameroon: A formative epidemiological and socioeconomic investigation in eleven rural fishing communities
Source: PLOS Glob Public Health. 2021 Oct 20;1(10):e0000007. doi: 10.1371/journal.pgph.0000007 (PMC10022362; doi:10.1371/journal.pgph.0000007)
Supplement: S1 Text — (DOCX) [file pgph.0000007.s002.docx]

**Close-ended structured questionnaire and in-depth interview/FGD guides**

1. **Close ended structured questionnaire (FGS symptoms, UGS and STI history, Water contact, Access to MDAs) – Administered directly in English with oral translations into Fulbe, Kotoko, Mosgum, and Arab**

Name of Community: ________________

Age (years): ________

Education: Informal education () Formal education () (specify) ______________

Marital Status: Single ( ) Married ( ) Separated ( ) Widowed ( )

No of years having lived in community _______

Previous community ________

**Questions on symptoms of urinary tract schistosomiasis; FGS; OR STI**

1. Do you see your menses? Yes ( ) No ( )
2. Did you observe/experience your menses within the last two weeks? Yes ( ) No ( )
3. Is your menses painful? ______ (pain during menstruation – always very painful, sometimes painful, normal) Irregular? ______ (every month?, not every month, stopped)
4. Do you have pain when urinating? Yes ( ) No ( )
5. Do you have difficulty in urinating (urine not coming out fluently)? Yes ( ) No ( )
6. Do you have sudden uncontrollable and unexpected urge to urinate and mostly cannot hold it? Even when you cough it comes out? Yes ( ) No ( )
7. Do you see blood in your urine? Yes () No ()
8. (If yes) When did you lastly see blood in your urine? ______ Always, sometimes, Once in a while
9. Do you sometimes have itching in your private part? Yes ( ) No ( )
10. How often do you experience this? Once a while ( ) frequently ( ) / When was the last time?
11. Do you have a feeling of burning within your private part? Yes ( ) No ( )
12. How often do you experience this? Once a while ( ) Frequently ( )
13. Do you sense a swelling/lumps within your private part? Yes ( ) No ( ) No response ( )
14. Do you have any discharge that comes from your vagina? Yes () No ( ) Does it have an ordure? ________ Do you see the colour? Yes ( ) No ( ) What Color is it? White; grey; green/yellow; brown
15. Do you think this is normal? Yes ( ) No ( ) Do not know (). When did you start observing the discharge? Date ___________________
16. After sexual intercourse do you have a discharge? Yes ( ) No ( ) Is it smelly? Yes ( ) No ( ) Do not know ( )
17. After or during sexual intercourse do you have pain? Yes ( ) No ( ) I do not know ( ) ; Do you have a bloody discharge? Yes ( ) No ( ) I do not know ( )
18. Have you had any miscarriages /pregnancies that passed? Yes ( ) No ( )
19. How many? ()
20. Do you have children? Yes() No()
21. What age is your last child? ()
22. Have you visited the clinic to complain about these issues? Yes ( ) No ( )
23. What you used/taken/done to treat any of these problems? ________ (Underline) Hospital or drug store/ local midwife/ traditional medicine/ prayers/ none of the above (what other? Specify) / nothing

**Water contact History**

1. Where do you fetch your household water? Lake; other source (name) ________
2. Do you fish in the lake? Yes (), No ()
3. Do you bathe in the lake? Yes (), No ()
4. What do you use the lake for? ()________

**MDAs**

1. Have you ever taken Praziquantel (distributed by…)? (Show drug example, and mentioning distributor who distributes in this community) Yes () No()
2. When was the last time you took Praziquantel? Within the last 6 months; within the last 2 years, never, cannot remember ________

1. **Interview Guides (FGS positive women and girls) – English Version**

Personal information

- Knowledge around FGS (Symptoms ; presentations /effects)
- Experiences with FGS symptoms like vaginal discharge, irregular menstruation, etc.
- Beliefs around FGS symptoms (STI related symptoms will be questioned and their similarities to FGS symptoms)
- Practices around FGS symptoms or effects complained or experienced
- Menstruation history (pains, irregularities, etc.) :perceptions/experiences
- Treatment sought for these symptoms (where, why, when…)
- Cost of treatment (financial/economic; psychosocial)
- Sexual history (post coital bleeding, pain during sex, etc.): perceptions/practices/ experiences (for married girls and women >15 )
- Fertility/sub-fertility - number of children, still-births, infertility, miscarriages ; personal perception/experience and general community perceptions/experiences around this
- Reproductive and productive activities (income generating and household chores/work) carried out and effect from disease

**Other community members and health workers**

- Personal information
- Knowledge around FGS
- Beliefs around FGS symptoms and effects (STI related symptoms will be questioned on and their similarities to FGS symptoms
- Practices around FGS symptoms and effects complained by wives /daughter / other female acquaintances
- Effect on Reproductive and productive activities (income generating and household chores/work) /

**Guides for FGD (group with women, group with young girls post- menarche, mixed group with health workers) – This guide is adaptable for each group mentioned here**

- Symptoms of STI, Symptoms of FGS
- Experiences with these symptoms
- Beliefs and practices around these symptoms (personal; family and immediate social network)
- Treatment seeking behaviors around these symptoms
- Stigma (self, family, health workers); other mental health discussions around effects.

**B. Guides d’entretien (femmes et filles positives du FGS) – French Version**

- Informations personnelles

- Connaissances autour du FGS

-Croit aux symptômes du FGS (les symptômes liés aux IST seront interrogés et leurs similitudes avec les symptômes du FGS

- Pratiques autour des symptômes FGS plaint ou expérimenté

- Pratiques autour des symptômes FGS ou des effets dénoncés ou expérimentés

-Histoire des menstruations (douleurs, irrégularités, etc.):perceptions/pratiques/expériences personnel et dénoncées par les membres de la communauté

- Antécédents sexuels (saignement post-coïtal, douleur pendant le sexe, etc.): perceptions/pratiques/expériences (Femmes (> 15 déjà maries ou sexuellement active)

- Reproduction (nombre d’enfants, naissances fixes, infertilité, fausses couches): perceptions/pratiques/expériences personnel ou des autres membres de la Communauté

- les activités de reproduction et production (activités génératrices de revenus) effectué et effet de la maladie

**Autres membres de la Communauté et personnels de la santé**

-Informations personnelles

-Connaissances autour de FGS

-Croit aux symptômes et aux effets de la FGS (les symptômes liés aux IST seront interrogés et leurs similitudes avec les symptômes du FGS

-Pratiques autour des symptômes et des effets FGS plaint par les épouses/fille/ autres connaissances féminines

- Effets sur les activités de reproduction et production (activités génératrices de revenues)

**Guide pour les FGDs (groupe des femmes, groupe des jeunes filles, groupe mixtes avec agents santé- Le guide est adaptable pour chaque groupe précisé**

- Symptômes de la STI, symptômes de FGS

- Expériences avec ces symptômes

- Croyances et pratiques entourant ces symptômes

- Traitement recherchant des comportements autour de ces symptômes

- Stigmatisation (auto, famille, travailleurs de la santé) ; Autre effets psychologique et sociale
