# Supplementary material for: Female Genital Schistosomiasis (FGS) in Cameroon: A formative epidemiological and socioeconomic investigation in eleven rural fishing communities
Source: PLOS Glob Public Health. 2021 Oct 20;1(10):e0000007. doi: 10.1371/journal.pgph.0000007 (PMC10022362; doi:10.1371/journal.pgph.0000007)
Supplement: Attachment — Submitted filename: Response to Reviewers.docx [file pgph.0000007.s003.docx]

**Rebuttal Letter**

**PGPH-D-21-00270**

**Female Genital Schistosomiasis (FGS) in Cameroon: A formative epidemiological and socioeconomic investigation in eleven rural fishing communities.**

**PLOS Global Public Health**

Dear Editor,

Thank you for considering our article, and for the constructive reviews. We have revised our manuscript, integrating all the points raised during the review process.
**Response to Reviewer #1:**

Dear Reviewer #1,

Thank you for your keen observations regarding our work and the important suggestions made for improving this manuscript. We have integrated all suggested changes.

- Line 88: We added this very precise, important and most recent systematic review and meta-analysis on the HIV/Schistosomiasis association. Thank you for this suggestion.
- Line 384-385 (now 369-371): The line of repetition through lines 369 – 371 has been deleted.
- Line 493-494: The mention of Figure 5 is a typing error, and has been changed to read “…The transport process as shown in Table 6…”
- Line 76-79: The references 7, 8, have been checked and corrected to provide better evidence for the underpinning statements.
  - Reference 7 was replaced with a more reflective reference - **King CH. Parasites and poverty: the case of schistosomiasis. Acta tropica. 2010;113(2):95-104** (now Ref 7 on line 76), - and the original Reference 7 (now Reference 8)- **Weiss MG. Stigma and the Social Burden of Neglected Tropical Diseases. PLOS Neglected Tropical Diseases. 2008;2(5):e237**, was moved to Line 76, where they both explain better the statement “[NTDs cause reduced ability to work] from both physical debilitating and detrimental mental health effects”.
  - Reference 8 (now Reference 9) - **WHO. Water, Sanitation & Hygiene. 2015–2020, A GLOBAL STRATEGY for accelerating and sustaining progress on Neglected Tropical Diseases Geneva: WHO; 2015 [Available from:** [**https://apps.who.int/iris/handle/10665/182735?locale-attribute=fr&**](https://apps.who.int/iris/handle/10665/182735?locale-attribute=fr&), was moved to Line 78, to provide better evidence for the statement “…most NTDs are not fatal”.
- Line 241-244: The highlighted errors with references (reference 42 and 43) are as a result of a bibliography transfer error within Endnote. These have been corrected.
  - References 42 has been removed and replaced with the references **- Cheesbrough M. Medical laboratory manual for tropical countries. Medical laboratory manual for tropical countries 1981;1. ; Campbell SJ, Stothard JR, O'Halloran F, Sankey D, Durant T, Ombede DE, et al. Urogenital schistosomiasis and soil-transmitted helminthiasis (STH) in Cameroon: An epidemiological update at Barombi Mbo and Barombi Kotto crater lakes assessing prospects for intensified control interventions. Infectious diseases of poverty. 2017;6(1):49**. These new references (now references 44 and 45) provide clearer evidence for the underpinned statement on use of Lugol’s iodine for the urine filtration technique and microscopy.
  - Reference 43 (now Reference 46) has been replaced with the reference - **WHO. Prevention and Control of Schistosomiasis and Soil Transmitted Helminths Geneva: WHO; 2002 [33/63]. Available from**[**http://apps.who.int/iris/bitstream/handle/10665/42588/WHO_TRS_912.pdf;jsessionid=297C20DDFCBFFF9F9AAE9DF120C0F834?sequence=1**](http://apps.who.int/iris/bitstream/handle/10665/42588/WHO_TRS_912.pdf;jsessionid=297C20DDFCBFFF9F9AAE9DF120C0F834?sequence=1)**,** to provide evidence for the underpinned statement on urine egg load definition and characterisation in urogenital schistosomiasis.

Thank you for this review and for recommending our manuscript for publication in PLoS Global Public Health Journal

**Response to Reviewer #2:**

Dear Reviewer #2,

Thank you for the keen observations and review, these have improved the content and reference body of our manuscript. We have considered and integrated all suggestions.

Line 97–8 **(now line 97 – 101):** The main objective of our study has been rephrased to be more precise and clearer. It now reads “*By shedding light on the epidemiological state and lived experiences of girls and women infected with FGS in Cameroon, our work sets to unveil the local importance of this disease, giving particular leeway into its psychosocial and economic effects, further expounding on the importance of prevention, accessible diagnostics and drug availability for FGS and reproductive health care as a whole.”*
Line 244 **(now line 245**): As suggested, the word ‘Colposcope’ has been included after ‘hand-held’.
Line 326-30 **(now line 334-338**): As suggested, the highlighted lines have been moved to after 342 (now 333). This section now begins with the sub-title “i. Demographic characteristics”
Line 329 **(now line 337):** After consideration, the statement here was reconsidered to now read “presence of *at least two of these* three main parameters”.
Line 334-5 **(now line 330-331):** As suggested, the last part of this sentence was rephrased to read “most of them (88.2%) living in a proximity of ≤100m to the lake”.
Line 335-42 **(now line 340- 347):** We added the heading “ii. Parasitological and clinical description” to this section, and added the lines under this.
Line 339 **(now line 344**): We removed the word ‘both’, as suggested.
Line 348 **(now line 332**): We moved Table 2 to ‘Demographic characteristics after line 335 (now 331) as suggested.
Line 531 **(now line 521):** We removed the statistical values ‘Logistic regression’, as suggested.
Line 532-5 **(522-524):** As suggested, we integrated the aspect of younger women having more acute infection and older women more severe chronic disease to read *“higher infection amongst this age group has been reported as reason of younger women having more acute infections, compared to older women who have more severe chronic disease from life-time duration of chronic sequelae of infection*”, with accompanying references to provide evidence.
Line 548 **(now line 542):** We remove the statistical value ‘Chi-squared’
Figure 3: We took out the error bars in Figure 3 as suggested..
Figure 4: Thank you for highlighting this.

**References**

In addition to Reviewer #1 and Reviewer #2 suggestions, and to provide most recent and clearer evidence to our statements within our manuscript, we have made the following additions and/or changes:

**Line 76, 78**: reference 7 (King CH. Parasites and poverty: the case of schistosomiasis. Acta tropica. 2010;113(2):95-104) and 9 (WHO. Water, Sanitation & Hygiene. 2015–2020, A GLOBAL STRATEGY for accelerating and sustaining progress on Neglected Tropical Diseases Geneva: WHO; 2015 [Available from: <https://apps.who.int/iris/handle/10665/182735?locale-attribute=fr&>), added on lines 76 and 78

**Line 550:** reference 7 (King CH. Parasites and poverty: the case of schistosomiasis. Acta tropica. 2010;113(2):95-104) and 52 (Pediatric Praziquantel Consortuim. Contract manufacturing agreement signed for production of new pediatric treatment for schistosomiasis 2021 [Available from: http//:<https://www.pediatricpraziquantelconsortium.org/>) added

**Line 557**: reference 53 (Hegertun IEA, Sulheim Gundersen KM, Kleppa E, Zulu SG, Gundersen SG, Taylor M, et al. S. haematobium as a common cause of genital morbidity in girls: a cross-sectional study of children in South Africa. PLoS neglected tropical diseases. 2013;7(3):e2104) added

**Line 620 – 622**: reference 66, 65, 66 added. (Countdown- Calling time on neglected tropical diseases. FGS Intervention manual. In: Countdown, editor.: Countdown; 2020. ; Bridges To Development / The FAST Package. Female Genital Schistosomiasis Training Competencies GSA: Bridges to Developement; 2021 [Available from: <https://www.learning.foundation/female-genital-schistosomiasis>; Bardosh K. Global aspirations, local realities: the role of social science research in controlling neglected tropical diseases. Infectious Diseases of Poverty. 2014;3(1):35.
